# Definition of the *jianfengling* species group of the ground beetle genus *Orthogonius* MacLeay (Coleoptera, Carabidae, Orthogoniini)

**DOI:** 10.3897/zookeys.615.9179

**Published:** 2016-09-07

**Authors:** Mingyi Tian, Thierry Deuve

**Affiliations:** 1 Department of Entomology, College of Agriculture, South China Agricultural University, Wushan, Guangzhou, 510640, China; 2 Muséum national d’Histoire naturelle, Département de Systématique & Évolution, Entomologie, C. P. 50, 57 rue Cuvier, F–5231 Paris cedex 05, France

**Keywords:** Carabids, Indo-Burma, new species, taxonomy, termitophilous

## Abstract

The *jianfengling* species group of the termitophilous carabid genus *Orthogonius* MacLeay, 1825 is defined and reviewed. This species group ranges from southern China, crossing Indochina and Myanmar to eastern India. To date, the *jianfengling* species group is composed of ten species, including six new species which are hereinafter described and illustrated: *Orthogonius wrasei* **sp. n.** (Myanmar), *Orthogonius bellus* **sp. n.** and *Orthogonius limbourgi* **sp. n.** (Vietnam), *Orthogonius politior* **sp. n.**, *Orthogonius aberlenci* **sp. n.** (Laos) and *Orthogonius meghalayaensis* **sp. n.** (India). Habitus, elytral apices and male genitalia of all species are illustrated. A key to species and a distribution map of *jianfengling* species group are provided.

## Introduction

Indo-Burma is one of the 25 biodiversity hotspots in the world for conservation priorities (Myers et al. 2000). It covers a large tropical area of more than two million square kilometers, from eastern India, through Myanmar to southern China to the east, and to northern of Malaya Peninsula to the south. The termitophilous ground beetles of the tribe Orthogoniini are very rich in species diversity in this area, represented by approximately 100 species (Tian and Deuve 2000, 2006, 2010, Tian et al. 2012).

The majority of orthogoniine species recorded in tropical Asia belongs to the prominent genus *Orthogonius* MacLeay, 1825. As the series works to understand the faunal composition and to set up a rational classification system of *Orthogonius*, the *lancangjiang* and *baconii* species groups have already been dealt with (Tian and Deuve 2013, 2016). In the present paper we define the *jianfengling* species group.

The *jianfengling* species group is comprised of members of large, polish, depressed and glabrous orthogoniine beetles. The first species of this group, *Orthogonius jianfengling* Tian & Deuve, was described in 2000 from Hainan Island, China. Then, *Orthogonius himalayicus* Tian & Deuve, 2005 (from Bhutan and Sikkim, India), *Orthogonius freyi* Tian & Deuve, 2006 (from Myanmar) and *Orthogonius duboisi* Tian & Deuve, 2006 (from Yunnan, China) were recorded respectively (Tian and Deuve 2005, 2006). To date, ten *Orthogonius* species are designed as members of the *jianfengling* species group, including six new species described below.

## Material and methods

All specimens for this study are dry and mounted materials. Dissection and observation of the specimens were made using a WILD M32 binocular microscope. Detailed descriptions are provided for the new species, while only diagnostic character states are given for the known species. Digital photographs were taken and processed in the same as in Tian and Deuve (2013).

Body length was measured from apex of right mandibles to apex of elytra; body width = width of elytra.

Abbreviations of measurements used in the text are as followings:

HL
 head length (from apex of right mandible to base of vertex) 


HW
 head width (maximum distance across head, including eyes) 


PL
 length of pronotum (measured from front to basal margins, through midline) 


PW
 width of pronotum (greatest width of pronotum) 


EL
 length of elytra (measured from base to apex of elytra, through suture) 


EW
 width of elytra (greatest transverse distance across both elytra) 


Abbreviations of collections mentioned in the text are as following

CDW
 Collection of David Wrase, Berlin 


CIB
 Collection of Ingo Brunk, Dresden 


CIRAD
 Agricultural Research for Development, Montpellier 


HNML
Natural History Museum, London



IRSNB
 Institut royal des Sciences naturelle de Belgique, Brussels 


MNHN
Muséum national d’Histoire naturelle, Paris 


NHMB
 Naturalhistorisches Museum, Basal 


NHMV
Natural History Museum of Vienna, Vienna 


SCAU
South China Agricultural University, Guangzhou 


## Taxonomic treatment

### Genus *Orthogonius* MacLeay, 1825

#### *jianfengling* species group

**Definition.** The *jianfengling* species group shares the following combination of morphological characteristics: (1) Medium to large sized; body elongate and more or less flat; polish and very smooth, strongly shiny, impunctate; (2) Labrum more or less concave or emarginate at frontal margin; ligula bisetose at apex; each of mentum and submentum bisetose; mentum edentate in most species (but toothed medially in *Orthogonius meghalayaensis* sp. n.); (3) Hind angle of pronotum rounded off; (4) Elytra well-bordered at base, nearly parallel-sided medially; striae shallow or deep, intervals convex, the 3^rd^ interval with three setiferous pores (but median one wanted in *Orthogonius freyi*), the 7^th^ interval not carinated; apex of elytra obliquely truncate, bisinuate or sinuate, outer apical angle rounded off, the inner apical angle various shaped, obtuse or rectangular, denticulate at tip or not; (5) Middle coxae setose medially in most species (but asetose in *Orthogonius bellus* sp. n., and *Orthogonius limbourgi* sp. n.); middle and hind tibiae slender, not dilated in male; femora moderately long; hind tibial spurs moderate in length, very sharp; the 4^th^ hind tarsomere bilobed; hind femora bisetose to quadrisetose posteriorly; tarsal claws especially the hind ones weakly pectinate; (6) Abdominal ventrite VII in male not emarginate at apical margin; and (7) Male genitalia not exposed; aedeagus short and stout, widely opened dorsally, apical lamella short; parameres normal for *Orthogonius*.

**Figures 1–2.**
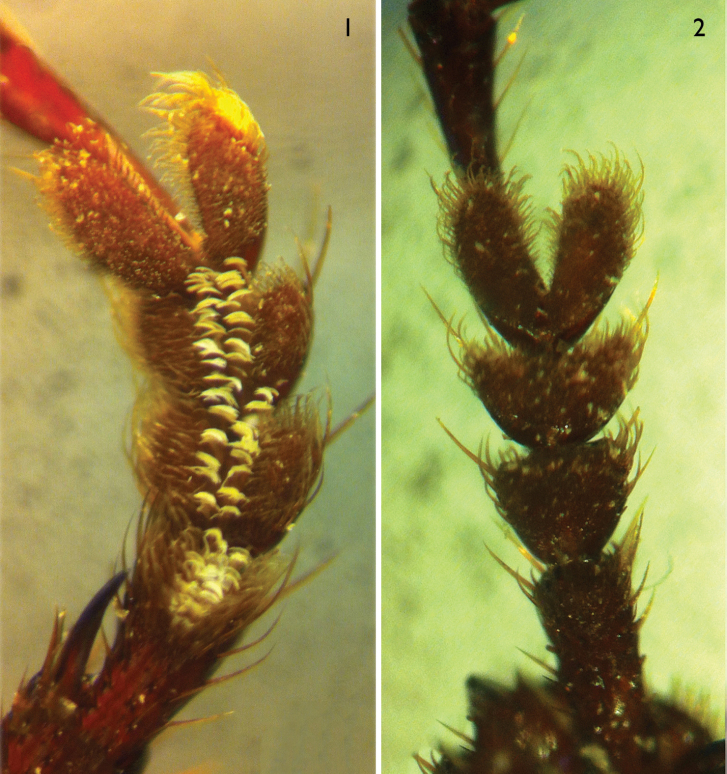
Protarsi of *Orthogonius limbourgi* sp. n. **1** male **2** female.

**Sexual dimorphism.** In male, 1^st^ (apical half) to 3^rd^ protarsomeres bearing two rows of spongy setae which are flat and located along median portion (Figs 1–2); ventrite VII not emarginated in both sexes.

**Figure 3.**
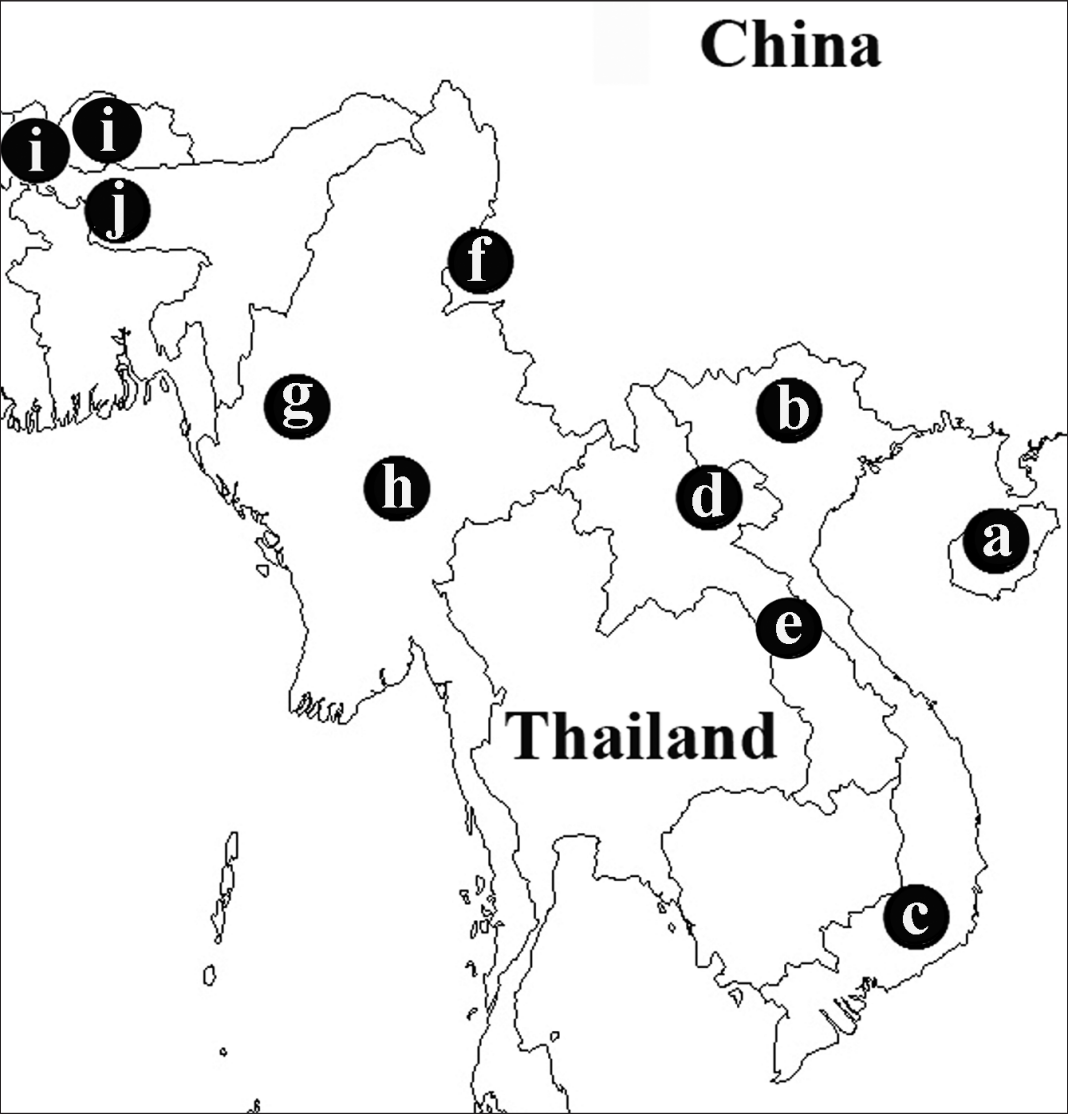
Distribution of the *jianfengling* species group **a** *Orthogonius jianfengling* **b** *Orthogonius limbourgi* sp. n. **c** *Orthogonius bellus* sp. n. **d** *Orthogonius politior* sp. n. **e** *Orthogonius aberlenci* sp. n. **f** *Orthogonius duboisi* **g** *Orthogonius wrasei* sp. n. **h** *Orthogonius freyi* **i** *Orthogonius himalayicus* **j** *Orthogonius meghalayaensis* sp. n.

**Distribution of the *jianfengling* species group.** The *jianfengling* species group are distributed in the following countries: China (*Orthogonius jianfengling* and *Orthogonius duboisi*), Vietnam (*Orthogonius bellus* sp. n. and *limbourgi* sp. n.), Laos (*Orthogonius politior* sp. n. and *Orthogonius aberlenci* sp. n.), Myanmar (*Orthogonius freyi* and *Orthogonius wrasei* sp. n., India (*Orthogonius meghalayaensis* sp. n., and *Orthogonius himalayicus*) and Bhutan (*Orthogonius himalayicus*) (Fig. 3).

##### Key to species of *jianfengling* species group

**Table:** 

1	Body large sized and depressed, polish, smooth and glabrous, ventrite VII in male not emarginate at apical margin, aedeagus not exposed	***jianfengling* species group**...**2**
–	Combination of other differences	**other species groups of *Orthogonius***
2	Prosternal process unbordered at apex	**3**
–	Prosternal process bordered at apex	**5**
3	Mentum with a median tooth, abdominal ventrite VII bearing three pairs of setae	***Orthogonius meghalayaensis* sp. n.**
–	Mentum edentate, abdominal ventrite VII bearing two pairs of setae	**4**
4	Head slender, labrum deeply emarginate at front, eyes less convex	***Orthogonius himalayicus***
–	Head stouter, labrum shallowly emarginate at front, eyes more convex	***Orthogonius politior* sp. n.**
5	Pronotum and elytra entirely reddish brown; scutellum widely obtuse; lateral expanded margins of pronotum distinctly reflexed	***Orthogonius jianfengling***
–	At least disc of pronotum light dark brown; scutellum narrowly obtuse; lateral expanded margins of pronotum either flat or slightly reflexed	**6**
6	Elytral interval 3 with only two setiferous pores, middle one wanted	***Orthogonius freyi***
–	Elytral interval 3 with three setiferous pores	**7**
7	Body brown or yellowish brown	**8**
–	Body black or dark brown	**10**
8	Middle coxae setose medially, apical inner angle of elytra obtuse, not denticulate at tip	***Orthogonius aberlenci* sp. n.**
–	Middle coxae asetose medially, apical inner angle of elytra nearly rectangular, distinctly denticulate at tip	**9**
9	Body more depressed, hind 3^rd^ tarsomere longer than 4^th^	***Orthogonius limbourgi* sp. n.**
–	Body less depressed, hind 3^rd^ tarsomere as long as 4^th^	***Orthogonius bellus* sp. n.**
10	Apex of elytron deeply sinuate, inner angle obtuse	***Orthogonius duboisi***
–	Apex of elytron shallowly sinuate, inner angle nearly rectangular	***Orthogonius wrasei* sp. n.**

###### Orthogonius jianfengling

Taxon classificationAnimaliaColeopteraCarabidae

Tian & Deuve, 2000

Figs 3a , 4 , 14

Orthogonius jianfengling Tian & Deuve, 2000: 298.

####### Length.

**Figures 4–7.**
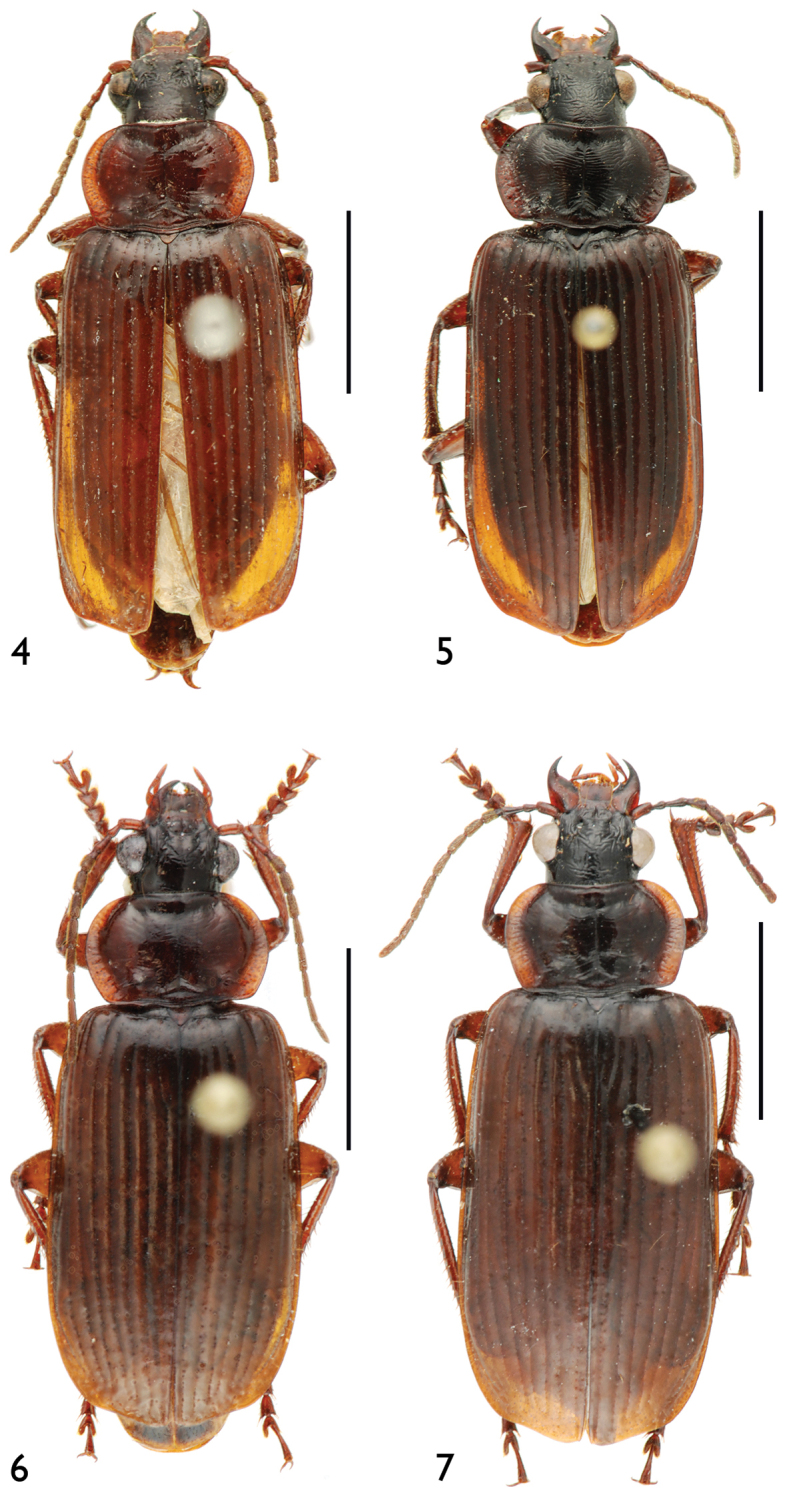
Habitus **4** *Orthogonius jianfengling* **5** *Orthogonius himalayicus* **6** *Orthogonius limbourgi* sp. n. **7** *Orthogonius politior* sp. n. Scale bar: 5.0 mm.

15.0–17.0 mm; width: 5.5–6.0 mm. Habitus as in Fig. 4.

####### Description.

Body elongate, strongly shiny, smooth and glabrous; head dark brown, pronotum, elytra and legs red brown, impunctate, microsculptural engraved meshes isodiametric. Head slightly longer than wide, HL/HW = 1.11; clypeus bisetose, labrum sex-setose, shallowly emarginate at apical margin; mentum edentate; each of mentum and submentum bisetose. Pronotum strongly transverse, PW/PL = 1.55, disc moderately convex, apical and basal margins well beaded, sides evenly expanded, widest at middle; base slightly wider than apex, lateral expanded margin well-defined, evenly and distinctly reflexed. Elytra elongate, and rather flat, EL/EW = 1.84, widest at about middle, parallel-sided at middle, basal border complete, apex bisinuate, inner angle blunt, not denticulate at tip (Fig. 14), the 3^rd^ interval with three setiferous pores. Legs slender, fore tibiae expanded at apex, with outer angle strongly protrude, outer margin smooth, sub-serrate; middle coxae setose medially, hind coxae smooth and glabrous, the 3^rd^ and 4^th^ hind tarsomeres subequal in length, the 4^th^ bilobed at apex; all tarsal claws weakly pectinate. Prosternal process bordered at apex.

Male: Unknown.

####### Remarks.

It is probably close to *Orthogonius limbourgi* sp. n., distinguished from the latter by its distinctly reflexed lateral expanded margins of pronotum (slightly reflexed in *Orthogonius limbourgi*), and setose middle coxae (asetose in *Orthogonius limbourgi*).

####### Material examined.

1 female, the holotype, “Hainan: Ledong Xian: Jianfengling, 15. VI. 1983, Gu Maobin leg.”, in SCAU; 1 female, a paratype, ibid. in MNHN.

####### Distribution.

China (Hainan Island) (Fig. 3a).

###### Orthogonius himalayicus

Taxon classificationAnimaliaColeopteraCarabidae

Tian & Deuve, 2005

Figs 3i , 5 , 15 , 24–25

Orthogonius himalayicus Tian & Deuve, 2005: 61.

####### Length.

19.0–20.0 mm; width: 6.5–7.0 mm (measurements in the original description were not correct). Habitus as in Fig. 5.

####### Description.

Head and pronotum black, antennae and elytra dark brown, slender and very shinny; body smooth and glabrous, head and pronotum impunctate, elytral odd intervals with a few, sparse punctures which are irregularly arranged; head intricately wrinkled; microsculptural engraved meshes densely isodiametric, faint on head and pronotum, clear on elytra. Head slightly longer than wide, HL/HW = 1.15–1.18; labrum deeply emarginate at front; eyes comparatively small, but prominent; mentum edentate and bisetose, submentum bisetose. Pronotum transverse, PW/PL = 1.91–1.94, rather flat, lateral expanded margin wide and slightly reflexed, widest at middle. Elytra elongate and rather slender, EL/EW = 1.74–1.77, parallel-sided at middle, moderately convex, widest slightly behind middle, apex obliquely truncate, inner angles roundly obtuse (Fig. 15); striae very deep, intervals distinctly convex, the 3^rd^ intervals generally with three setiferous pores. Legs moderately long, middle coxae setose, the 3^rd^ and 4^th^ hind tarsomeres subequal in length, all tarsal claws weakly pectinate. Prosternal process unbordered at apex. Male genitalia (Figs 24–25) with the median lobe of aedeagus comparatively small and short, middle portion convex, apex pointed in lateral view, turning to right, obtuse dorsally.

####### Remarks.

Differs from other congeners by its deeply concaved labrum and broadly obtuse inner apex of elytron.

####### Material examined.

1 male, the holotype: “British Bootang, L. Durel, 1898”, in MNHN; 2 males, paratypes, “Pedong, A. Desgodins”; 1 male and 2 females, paratypes, “British Bootang, Maria Basti. L. Durel”; 1 male, a paratype: “Sikkim” only; 1 female, a paratype: “Museum Paris, Bhutan. R. Oberthür, 1900”, all in MNHN, except one male in SCAU.

####### Distribution.

Bhutan and Sikkim (Fig. 3i).

###### Orthogonius limbourgi sp. n.

Taxon classificationAnimaliaColeopteraCarabidae

http://zoobank.org/107BEE2C-7B57-4A69-9CF4-E7B8FC708376

Figs 1–2 , 3b , 6 , 16 , 26–27

####### Holotype.

Male, label “Coll. I.R.Sc.N.B./ Vietnam: Tam Dao NP, 25-28. VII, 2010, light trap, Leg. J. Constant & P. Limbourg; I.G. 31.668”, in IRSNB.

####### Paratypes.

1 female, “Coll. I.R.Sc.N.B./ Vietnam: Cuc Phuong, Day coll. NG/IG, 31.668, 11-18. VIII, 2010, Leg J. Constant & P. Limbourg”, in IRSNB; 25 males & 14 females, “Coll. I.R.Sc.N.B./ Vietnam: Cuc Phuong N. P., 20.19’00” N, 105.36’30” E, 19-23. VII, 2011, night collecting, Leg J. Constant & P. Bresseel; I.G. 31.933”, in IRSNB, MNHN and SCAU, respectively; 1 male, “N. Vietnam, Tamdao NP env. 1000 m, VI. 2012, ex coll. A. Popadic”, in CIB; 2 females, in CIB and MNHN respectively.

####### Diagnosis.

A medium-sized species, light dark brown in general, with rather flat and slender body which is smooth and glabrous, and asetose midcoxae and well bordered prosternal process.

####### Description.

Length: 15.0–16.0 mm; width: 6.0–6.5 mm. Habitus as in Fig. 6.

Body elongate, strongly shiny, smooth and glabrous. Head and disc of pronotum dark brown; elytra, lateral expanded margins of pronotum, mouthparts, antennae, legs and ventral surface reddish brown; whole body dark brown in several specimens. Surface impunctate, head moderately wrinkled on frons, smooth on vertex and pronotum. Microsculptural engraved meshes isodiametric on elytra, indistinct on head and pronotum.

Head longer than wide, HL/HW = 1.12–1.14, eyes small but prominent, frons and vertex moderately convex, frontal impressions large and deep; clypeus bisetose, basal portion even; labrum sex-setose, shallowly emarginate at front margin; palps slender, subcylindrical, the 3^rd^ maxillary palpomere as long as the 4^th^ which is glabrous, the 3^rd^ palpomere with two short setae at apex; the 2^nd^ labial palpomere slightly longer than the 3^rd^, bisetose in inner margin, with several additional setae at subapex and apex, the 3^rd^ labial palpomere with a few setae at basal part; ligula small, bisetose at apex; mentum edentate; each of mentum and submentum bisetose (but an additional seta on the left of mentum in male), palpiger asetose. Antennae moderately long, extending over base of elytra; pubescent from basal 1/3 of the 4^th^ antennomere, slightly expanded at pubescent portion of the 4^th^; the 3^rd^ antennomere almost as long as the 4^th^.

Pronotum strongly transverse, PW/PL = 1.59–1.62, disc moderately convex, apical and basal margins well beaded, sides evenly expanded, widest at middle; base slightly wider than apex, bisinuate on base, slightly concave on apical margin; hind angle rounded off; lateral expanded margin well defined, evenly and indistinctly reflexed; both transversal impressions distinct, basal foveae small and deep.

Elytra elongate, and rather flat; EL/EW = 1.64–1.65; widest at about middle, sides parallel at middle, basal border complete, apex bisinuate, inner angle broad, with a small denticle (Fig. 16); striae moderately deep, intervals convex, odd and even intervals subequal in width in middle, the 3^rd^ interval with three setiferous pores; the 7^th^ interval simple.

Legs slender, fore tibiae expanded at apex, with outer angle strongly protrude, outer margin serrate; hind femora rather slender, with three posterior setae; middle coxae setose, hind coxae smooth and glabrous; middle tibiae quite straight, slightly expanded at apex; hind tibiae slightly expanded at apex, apical spurs long and sharp, the 3^rd^ hind tarsomere slightly longer than the 4^th^ which bilobed at apex; all tarsal claws weakly pectinate.

Prosternal process well bordered at apex. Abdominal ventrite VII of male complete; ventrite VII with two pairs of setae on either side of apical margin in both male and female.

Male genitalia: Stout, dorsal opening wide and long, base dilated, gently bent ventrally towards apex, which almost pointed in lateral view; in dorsal view, not contracted before apical lamella, which short and broad, as long as wide, surface granulated.

####### Remarks.

It is allied to *Orthogonius politior* sp. n., but its clypeus bisetose (quadrisetose in *Orthogonius politior*), prosternal process well-bordered at apex (unbordered in *Orthogonius politior*), and the apical lamella of aedeagus shorter and broad at apex (longer and narrow in *Orthogonius politior*).

####### Etymology.

In honor of P. Limbourg (Brussels), one of the holotype collectors.

####### Distribution.

Northern Vietnam (Fig. 3b).

###### Orthogonius politior sp. n.

Taxon classificationAnimaliaColeopteraCarabidae

http://zoobank.org/32834F91-D7F4-4801-B957-936966D44D1F

Figs 3d , 7 , 17 , 28–30

####### Holotype.

Male, “Laos-NE: Houa Phan Province, 20.13N/104.00E, Phou Pane Mt., 1350-1500 m, 1-16. VI. 2009, M. Brancucci leg.”, “NHMB Basel, NMP Prague, Laos 2009 expedition: M. Brancucci, M. Geiser, Z. Kraus, D. Hauck, V. Kuban”, in NHMB.

####### Diagnosis.

A species similar to but larger than the above one, with also a rather flat and slender body, but having an unbordered prosternal process, a quadrisetose clypeus and setose midcoxae.

####### Description.

Length: 19.0 mm; width: 6.8 mm. Habitus as in Fig. 7.

Body elongate, strongly shiny, smooth and glabrous. Head and disc of pronotum dark brown; elytra, lateral expanded margins of pronotum, mouthparts, antennae, legs and ventral surface reddish brown. Head and pronotum impunctate, elytral odd intervals with a few punctures; head moderately wrinkled; pronotum and elytra largely smooth. Microsculptural engraved meshes isodiametric on elytra, rather transverse on pronotum, indistinct on head.

Head longer than wide, HL/HW = 1.09, eyes small but prominent, frons and vertex convex, frontal impressions large and deep; clypeus quadrisetose in male, basal portion even; labrum sex-setose, shallowly emarginate at apical margin; palps slender, subcylindrical, the 3^rd^ maxillary palpomere as long as the 4^th^ which glabrous, the 3^rd^ maxillary palpomere with two short setae at apex; the 2^nd^ labial palpomere slightly longer than the 3^rd^, bisetose on inner margin, with several additional setae at subapex and apex, the 3^rd^ labial palpomere with a few setae at basal part; ligula small, bisetose at apex; mentum edentate; each of mentum and submentum bisetose (but a third seta on the left of mentum in the holotype), palpiger asetose. Antennae moderately long, extending over base of elytra; pubescent from basal 1/3 of the 4^th^ antennomere, slightly expanded at pubescent portion of the 4^th^; the 3^rd^ as long as the 4^th^.

Pronotum strongly transverse, PW/PL = 1.60, disc quite flat, apical and basal margins well beaded, sides evenly expanded, widest at middle; base wider than apex, bisinuate on base, slightly concave on apical margin; lateral expanded margin well defined, evenly and distinctly reflexed; both transversal impressions distinct, basal foveae small and deep.

Elytra elongate-ovate, EL/EW = 1.75; widest at about middle, sides parallel at middle, basal border complete, apex bisinuate, inner angle broad, but shortly denticulate (Fig. 17); striae moderately deep, intervals convex, subequal in width of odd and even intervals in middle, the 3^rd^ interval with three setiferous pores; the 7^th^ interval normal.

Legs rather slender, hind femora rather slender, with three posterior setae; fore tibiae expanded at apex, with outer angle strongly protrude, outer margin serrate; middle coxae setose, hind coxae smooth and glabrous; middle tibiae quite straight, slightly expanded at apex; hind tibiae slightly expanded at apex, apical spurs long and sharp, the 3^rd^ hind tarsomere slightly longer than the 4^th^ which bilobed at apex; all tarsal claws weakly pectinate.

Prosternal process unbordered at apex.

Male genitalia (Figs 28–30): Median lobe of aedeagus stout, dorsal opening wide and long, base dilated, gently bent ventrally towards apex, which almost pointed in profile; in dorsal view, median lobe distinctly contracted before apical lamella which short and thin, longer than wide.

Female: Unknown.

####### Remarks.

It is very similar to *Orthogonius limbourgi*, with the differences mentioned above.

####### Etymology.

Referred to its polished body.

####### Distribution.

Laos (Fig. 3d).

###### Orthogonius aberlenci sp. n.

Taxon classificationAnimaliaColeopteraCarabidae

http://zoobank.org/15B791FA-FBB3-4DD9-8349-3BE664F7228D

**Figures 8–11.**
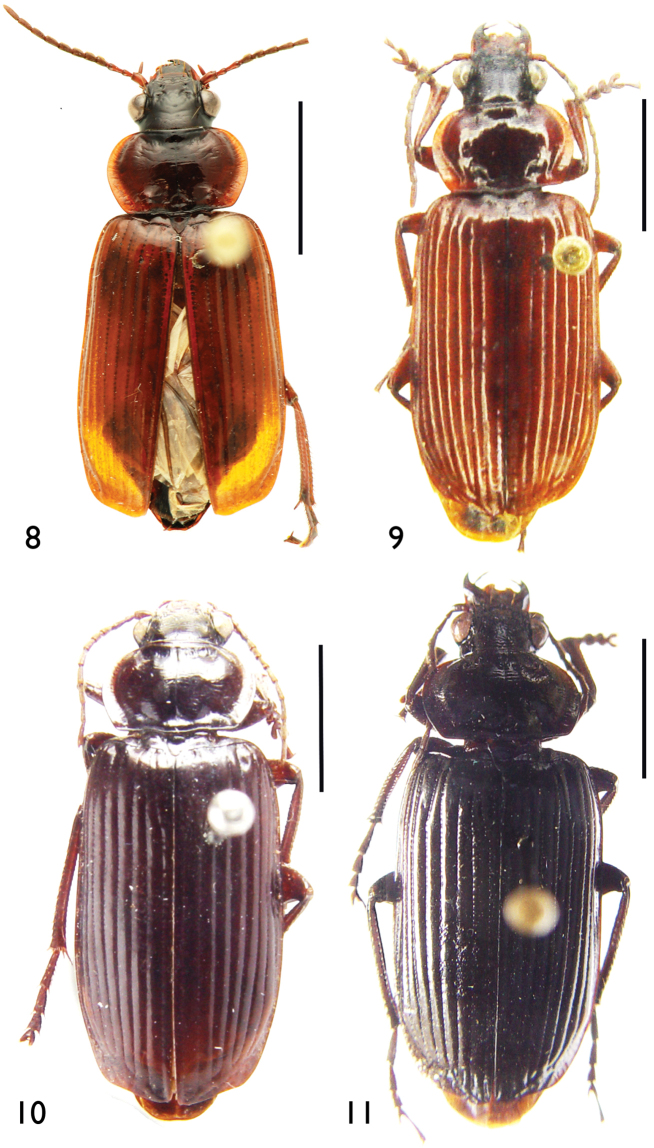
Habitus **8** *Orthogonius aberlenci* sp. n. **9** *Orthogonius bellus* sp. n. **10** *Orthogonius freyi* **11** *Orthogonius duboisi*. Scale bar: 5.0 mm.

Figs 3e , 8 , 18 , 31–32

####### Holotype.

Male, label: “Laos: Province de Khammouane, rivière Hin Boun, Ban Nathan, campe de l’Agame, 17°59.773'N / 104°49.395'E, V. 2012, Piège Lumineux, IBCFL, Opération Canopée, H.-P. Aberlenc leg.”, in MNHN.

####### Paratypes.

1 male & 3 females, in MNHN, SCAU and CIRAD (Montpellier).

####### Diagnosis.

A medium-sized and stout species, with a shiny, smooth, and glabrous body which is brownish in general, and having an edentate mentum, a well bordered prosternal process and setose midcoxae.

####### Description.

Length: 15.0–16.5 mm; width: 6.0–6.5 mm. Habitus as in Fig. 8.

Middle sized, stout, strongly shiny, smooth and glabrous. Head black; palps, pronotum, elytra, the 1^st^–3^rd^ antennomeres, legs and ventral surface brownish; labrum, clypeus, the 4^th^ to 11^th^ antennomeres dark brown. Head and pronotum impunctate, elytral odd intervals with a few fine and feeble punctures. Microsculptural engraved meshes isodiametric on elytra, indistinct on head and pronotum.

Head as long as wide, eyes moderate and prominent, frons and vertex convex, frontal impressions large and deep, extending posteriorly to the level of anterior supraorbital pores; clypeus bisetose, basal portion decorated by three short, longitudinal furrows which crossing base of clypeus and joining frontal impressions on either sides, and against frons medially; labrum sex-setose, moderately emarginate at apical margin; palps stout, subcylindrical, the 3^rd^ maxillary palpomere slightly shorter than the 4^th^, glabrous on the 4^th^, the 3^rd^ with three short setae at apex; the 2^nd^ labial palpomere slightly longer than the 3^rd^, bisetose on inner margin, with several additional setae at subapex and apex, the 3^rd^ labial palpomere with a few setae at basal part; ligula small, bisetose at apex; mentum edentate; each of mentum and submentum bisetose, setae subequal in length, palpiger asetose. Antennae moderately long, extending over base of elytra; pubescent from basal 1/3 of the 4^th^ antennomere, where evidently expanded; the 3^rd^ antennomere almost as long as the 4^th^, the 1^st^ with a long seta at subapex.

Pronotum strongly transverse, PW/PL = 1.65, disc quite flat, apical and basal margins well beaded, sides evenly expanded, widest at middle; base wider than apex, bisinuate on base, slightly and widely concave on apical margin; lateral expanded margins well defined, narrow, evenly and distinctly reflexed; fore transversal impression faint, hind one distinct, basal foveae small but deep.

Elytra elongate, EL/EW = 1.70–1.75; widest at about middle, almost parallel-sided at middle, basal border complete, apex widely and nearly obliquely truncated on outer margin, then reversely truncated nearly the tip, inner angle nearly rectangular, not denticulate (Fig. 18); striae moderately deep, intervals convex, intervals subequal in width in middle, the 3^rd^ interval with three setiferous pores; the 7^th^ interval normal.

Legs moderately long, hind femora moderately expanded, with three posterior setae in male and four or five in female; fore tibiae expanded at apex, with outer angle shortly protruded, outer margin sub-serrate; middle coxae glabrous in holotype, but with one to three setae in median portion in other specimens, hind coxae smooth and glabrous; middle tibiae not modified in male, quite straight, serrate, gradually expanded towards apex; hind tibiae slightly expanded at apex, apical spurs very long and sharp, the 1^st^ hind tarsomere distinctly longer than the 2^nd^, the 3^rd^ as long as the 4^th^ which deeply and symmetrically bilobed at apex; all tarsal claws weakly pectinate.

Prosternal process bordered at apex. Abdominal ventrite VII of male complete, with two pairs of setae on either side of apical margin in both male and female.

Male genitalia (Fig. 31–32): Short and stout, dorsal opening wide and long, somewhat expanded medially, gently bisinuate ventrally towards apex, which short and blunt in lateral view; apical lamella short and small, not parallel-sided, wider than long, apex broad.

####### Remarks.

It is probably allied to *Orthogonius limbourgi* sp. n. as they have similar structure of aedeagus. But it is easily separated from *Orthogonius limbourgi* and *Orthogonius politior* sp. n. by its short and more convex body, and short apical lamella of the aedeagus.

####### Etymology.

The name of this new species is in honor of the collector, Henri-Pierre Aberlenc (CIRAD, Montpellier), an excellent entomologist and a friend of the second author.

####### Distribution.

Laos (Fig. 3e).

###### Orthogonius bellus sp. n.

Taxon classificationAnimaliaColeopteraCarabidae

http://zoobank.org/AD1E656E-12F7-4F88-AD5C-ACF59BA56445

Figs 3c 9, 19, 36–37

####### Holotype.

Male, “S. Vietnam, 40 km NW An Khe, Buon Luoi, 620-750 m”, “14.10 N / 108.30 E, 28. III-12.IV. 1995, Pacholatko & Dembicky”, “Mus. Wien.”

####### Paratype.

1 male, ibid. in NHMV.

####### Diagnosis.

A fairly large species, with a yellowish and slender body which is less depressed, but strongly shiny, smooth and glabrous, having a bordered prosternal process, an edentate mentum and asetose midcoxae.

####### Description.

Length: 18.5 mm; width: 7.0 mm. Habitus as in Fig. 9.

Body elongate, strongly shiny, smooth and glabrous. Yellowish to brown, except head which dark brown. Head and pronotum impunctate, elytral odd intervals with a few punctures. Microsculptural engraved meshes isodiametric on head and elytra, transverse on pronotum.

Head longer than wide, HL/HW = 1.12, eyes small but prominent, frons and vertex convex, frontal impressions deep and long, extending beyond the level of fore supraorbital setae; clypeus bisetose, basal portion of surface uneven; labrum sex-setose, faintly emarginate at apical margin; palps slender, subcylindrical, the 3^rd^ maxillary palpomere as long as the 4^th^ which glabrous; the 3^rd^ maxillary palpomere with two short setae at apex; the 2^nd^ labial palpomere slightly longer than the 3^rd^, bisetose on inner margin, with several additional setae at subapex and apex; the 3^rd^ labial palpomere with a few setae at basal part; ligula small, bisetose at apex; mentum edentate; each of mentum and submentum bisetose, palpiger asetose. Antennae rather long, extending basal one third of elytra; pubescent from basal one third of the 4^th^ antennomere, slightly expanded at pubescent portions; the 3^rd^ antennomere almost as long as the 4^th^.

Pronotum strongly transverse, PW/PL = 1.64, disc moderately convex, apical and basal margins well beaded, sides evenly expanded, widest at middle; lateral expanded margin well defined and reflexed, surface uneven; both transversal impressions well marked, basal foveae small and deep.

Elytra elongate-ovate, EL/EW = 1.69; widest at about apical one third of elytra, sides parallel at middle, basal border complete, apex broadly sinuate, inner angle nearly rectangular (Fig. 19); striae deep, intervals distinctly convex, subequal in width with each other at middle, the 3^rd^ interval with three setiferous pores.

Legs rather slender, fore tibiae slightly expanded at apex, outer angle distinctly protruding and sharp, outer margin somewhat sub-serrate; middle and hind coxae smooth and glabrous; middle tibiae quite straight, slightly expanded at apex; hind tibiae hardly expanded at apex, apical spurs long and sharp; the 3^rd^ hind tarsomere as long as the 4^th^ which deeply bilobed at apex; claws weakly pectinate.

Prosternal process faintly bordered at apex.

Male genitalia (Figs 36–37): Stout, slightly sinuate at ventral side, dorsal opening wide, apex densely spinulate, apical lamella very short, broad, much wider than long.

Female: Unknown.

####### Remarks.

Probably close to *Orthogonius limbourgi* sp. n., but its body less depressed, and hind 3^rd^ tarsomere as long as 4^th^ (versus in *Orthogonius limbourgi*).

####### Etymology.

“*Bellus*”, a Latin word meaning beautiful, refering to the charmof this species.

####### Distribution.

Southern Vietnam (Fig. 3c).

###### Orthogonius freyi

Taxon classificationAnimaliaColeopteraCarabidae

Tian & Deuve, 2006

Figs 3h , 10 , 20 , 36–37

Orthogonius freyi Tian & Deuve, 2006: 126.

####### Length.

17.0 mm; width: 6.5 mm. Habitus as in Fig. 10.

####### Description.

Dark brown to black, body rather slender and depressed, smooth and glabrous, microsculptural engraved meshes isodiametric on head, pronotum and elytra. Head longer than wide, HL/HW = 1.16, eyes rather small; labrum straight at frontal margin, sex-setose, clypeus bisetose; each of mentum and submentum each with a pair of setae. Pronotum strongly transverse, PW/PL = 1.60, widest a little before middle, lateral expanded margins well-marked, almost flat; elytra elongate-ovate, EL/EW = 1.73, convex, base well-bordered, parallel-sided, striae deep, intervals strongly convex, apex faintly sinuate, inner angle nearly rectangular, distinctly denticulate at tip (Fig. 20); the 3^rd^ interval with two well-marked setiferous pores, median one wanted. Legs moderately long, the 4^th^ hind tarsomere shorter than the 3^rd^, lobes of 4^th^ tarsal emarginations half long as the tarsomere; tarsal claws weakly pectinate. Prosternal process well-bordered at apex; abdominal ventrite VII not emarginate at apical margin in male. Male genitalia (Figs 36–37) stout and short, apical lamella short, as wide as long at apex, tip almost truncated.

####### Remarks.

Differs from other members of this species group by its labrum straight at front, and 3^rd^ elytral interval with only two setiferous pores.

####### Material examined.

The type series only: 1 male, holotype, “Birmah Momeit”, “Doherty” “Frey Coll. 1905.100”, “60827” and “*Orthogonius* sp.” (By Andrewes); 1 female, a paratype, ibid, in NHML.

####### Distribution.

Myanmar (Fig. 3h).

###### Orthogonius duboisi

Taxon classificationAnimaliaColeopteraCarabidae

Tian & Deuve, 2006

Figs 3f , 11 , 21 , 38–39

Orthogonius duboisi Tian & Deuve, 2006: 124

####### Length.

19.0 mm; width: 7.0 mm. Habitus as in Fig. 11.

####### Description.

Dark brown, shiny, glabrous and smooth, but faint striate on head and pronotum; microsculptural engraved meshes isodiametric. Head slightly longer than wide, HL/HW = 1.10, eyes moderately sized, prominent, labrum slightly emarginate at frontal margin, clypeus bisetose; antennae backwardly exceeding elytral humeri. Pronotum transverse, PW/PL = 1.77, widest a little before middle, lateral expanded margin quite wide, slightly reflexed. Elytra elongate-ovate, rather flat, base well-bordered, parallel-sided medially, apex moderately sinuate, outer angle indistinct, inner angle large and obtuse, not denticulate (Fig. 21); striae deep, intervals slightly convex, subequal in width at middle; the 3^rd^ interval with three setiferous pores. Hind tibial spurs quite short, but sharp; the 4^th^ tarsomere shorter than the 3^rd^, deeply emarginate at apex, with lobes nearly half as long as joint. The prosternal process bordered at apex. Male genitalia (Figs 38–39): Short and stout, apex broad, the apical lamella very short and broad.

Female: Unknown.

####### Remarks.

It is similar to *Orthogonius freyi*, but its inner apical angle of elytra is obtuse and not denticulate at tip (versus in *Orthogonius freyi*), and the male aedeagus is stouter.

####### Material examined.

1 male, the holotype, “CHINE-Yunnan, Tongbiguan, 24°36'N, 97°35'E, 1180 m”, “14. VI. 2001, Deuve, Mantilleri, Rougerie & Tian leg.”, in SCAU.

####### Distribution.

China (Yunnan).

###### Orthogonius meghalayaensis sp. n.

Taxon classificationAnimaliaColeopteraCarabidae

http://zoobank.org/7151FBFF-2EDC-44EA-AF90-C54217557D6A

**Figures 12–13.**
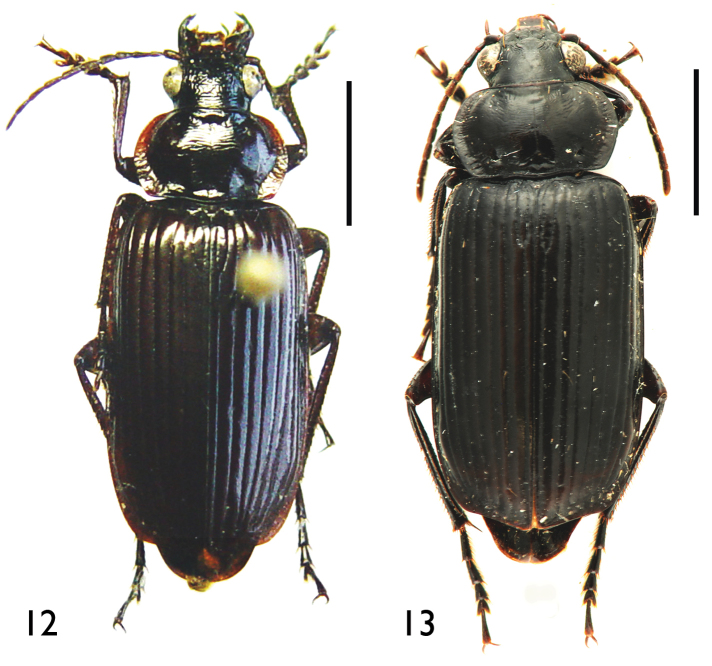
Habitus **12** *Orthogonius meghalayaensis* sp. n., holotype, male **13** *Orthogonius wrasei* sp. n., paratype, female. Scale bar: 5.0 mm.

Figs 3j , 12 , 22 , 40–41

####### Holotype.

Male, “NE India: Meghalaya, W. Garo Hills, Nokrek National Park, ca 1000 m”, “25.29.06 N / 90.19.05 E, 9-17. V. 1996, leg. Jendek & Sausa”, “Mus. Wien.”

####### Paratype.

1 female, ibid, in NHMV.

####### Diagnosis.

A dark brown or black species, having a elongate body, a deeply emarginate labrum, an edentate mentum, a well bordered prosternal process, setose midcoxae and a 6-setose ventrite VII.

####### Description.

Length: 18.5–19.0 mm; width: 6.5 mm. Habitus as in Fig. 12.

Body elongate, shiny. Head and disc of pronotum black to dark brown; elytra, lateral expanded margins of pronotum, mouthparts, antennae, legs and ventral surface reddish dark brown. Female darker than male. Head and pronotum impunctate, elytral odd intervals with a few punctures; head densely and moderately wrinkled and intricate; pronotum transversally striate, elytra smooth. Microsculptural engraved meshes isodiametric on elytra, rather transverse on head and pronotum.

Head longer than wide, HL/HW = 1.04, eyes small but prominent, frons and vertex convex, frontal impressions deep; clypeus bisetose, basal portion of surface uneven; labrum sex-setose, deeply emarginate at apical margin; palps slender, subcylindrical, the 3^rd^ maxillary palpomere as long as the 4^th^ which glabrous, the 3^rd^ maxillary palpomere with two short setae at apex; the 2^nd^ labial palpomere slightly longer than the 3^rd^, bisetose on inner margin, with several additional setae at subapex and apex, the 3^rd^ labial palpomere with a few setae at basal part; ligula small, bisetose at apex; mentum with a median tooth, but not well-defined; each of mentum and submentum bisetose, palpiger asetose. Antennae moderately long, extending over base of elytra; pubescent from basal one third of the 4^th^ antennomere, slightly expanded at pubescent portion; the 3^rd^ antennomere almost as long as the 4^th^.

Pronotum strongly transverse, PW/PL = 1.60, disc moderately convex, apical and basal margins well beaded, sides evenly expanded, widest at middle; lateral expanded margins well defined, flat and not reflexed, surface uneven; both transversal impressions distinct, basal foveae small and deep.

Elytra elongate ovate, EL/EW = 1.67, widest at about middle, sides parallel at middle, basal border complete, apex broadly sinuate, inner angle broadly acute (Fig. 22); striae shallow, intervals rather flat or slightly convex, subequal in width with each other in middle, the 3^rd^ interval with three setiferous pores; the 7^th^ interval normal.

**Figures 14–23.**
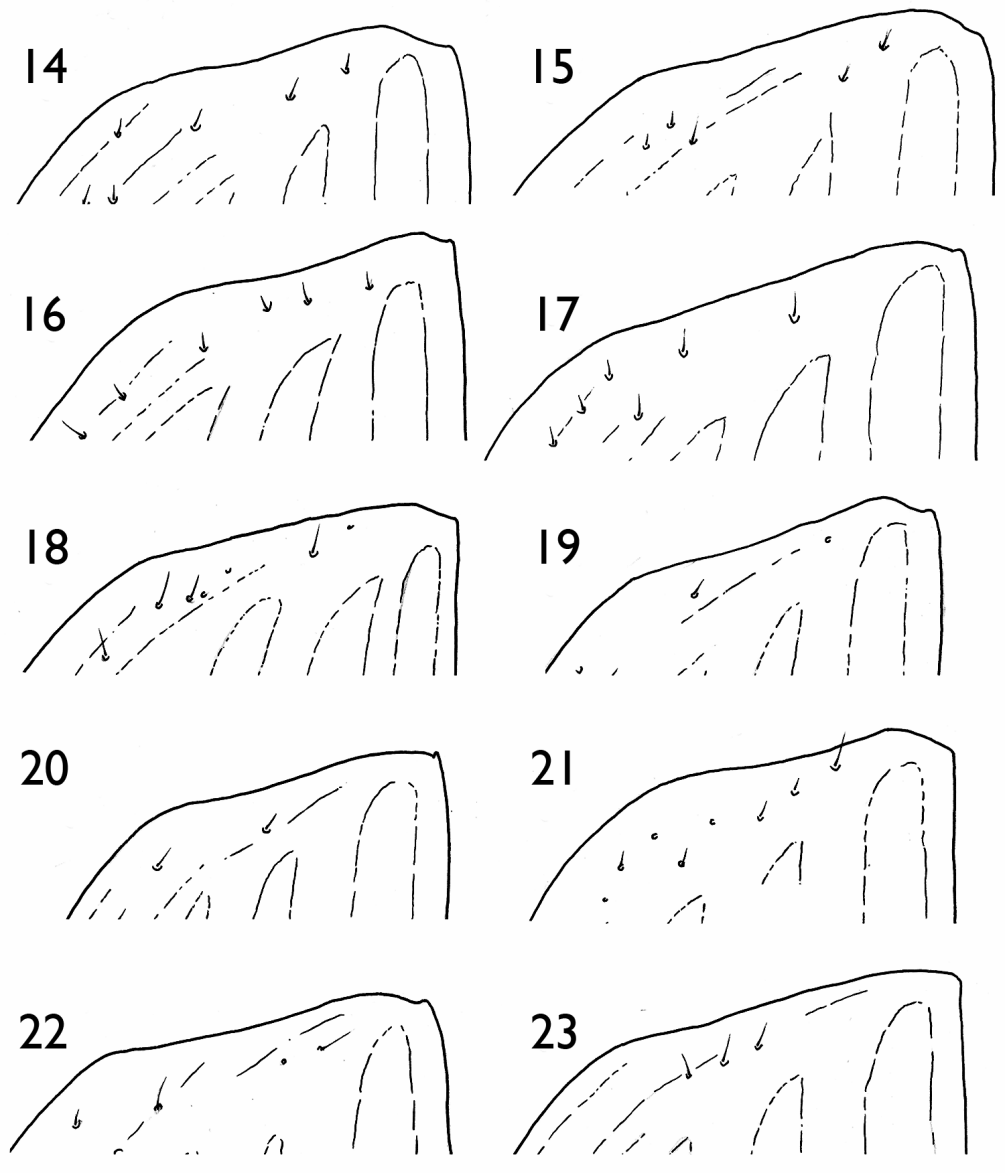
Apex of right elytron **14** *Orthogonius jianfengling* **15** *Orthogonius himalayicus* **16** *Orthogonius limbourgi* sp. n. **17** *Orthogonius politior* sp. n. **18** *Orthogonius aberlenci* sp. n. **19** *Orthogonius bellus* sp. n. **20** *Orthogonius freyi* **21** *Orthogonius duboisi* **22** *Orthogonius meghalayaensis* sp. n. **23** *Orthogonius wrasei* sp. n.

Legs rather slender, fore tibiae slightly expanded at apex, outer angle distinctly protrude, outer margin somewhat sub-serrate; middle and hind coxae smooth and glabrous; middle tibiae quite straight, slightly expanded at apex; hind tibiae hardly expanded at apex, apical spurs long and sharp, the 3^rd^ hind tarsomere much longer than the 4^th^ which deeply emarginate at apex (lobes half as long as the joint); claws weakly pectinate.

Prosternal process unbordered at apex. Ventrite VII with three pairs of setae on either side of apical margin in both sexes.

Male genitalia (Figs 40–41): Short and stout, nearly straight ventrally, dorsal opening very large, almost extending to base; apical lamella short, contracted towards apex, slightly wider than long.

**Figures 24–30.**
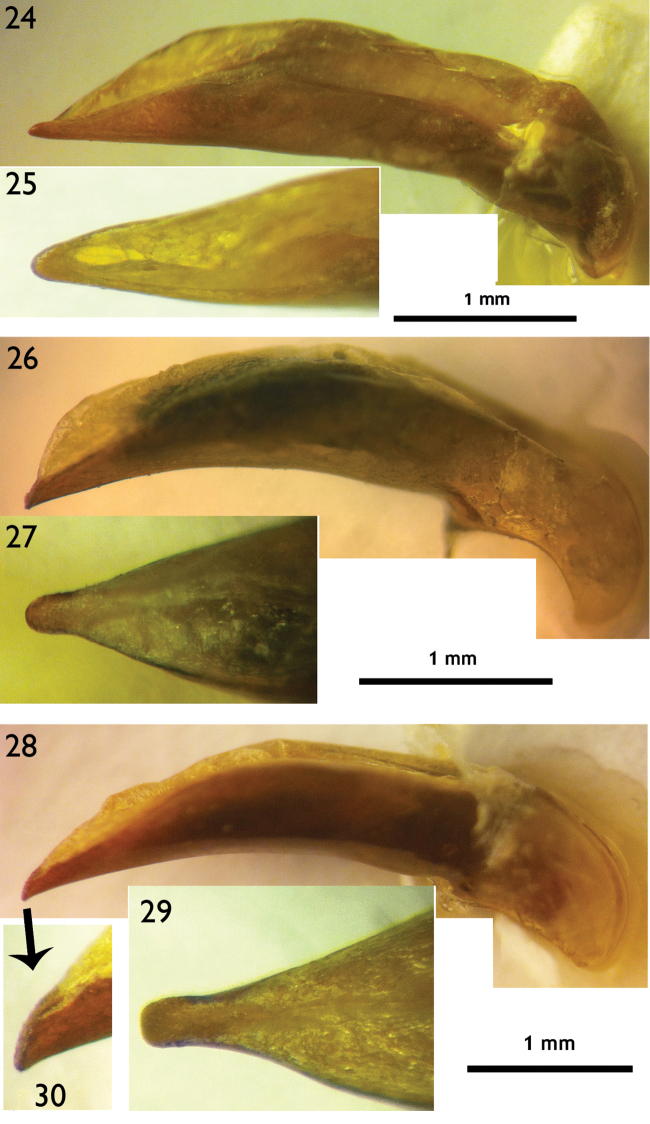
Male genitalia (right lateral view and dorsal view for apical lamella) **24–25** *Orthogonius himalayicus* **26–27** *Orthogonius limbourgi* sp. n. **28–30** *Orthogonius politior* sp. n.

**Figures 31–37.**
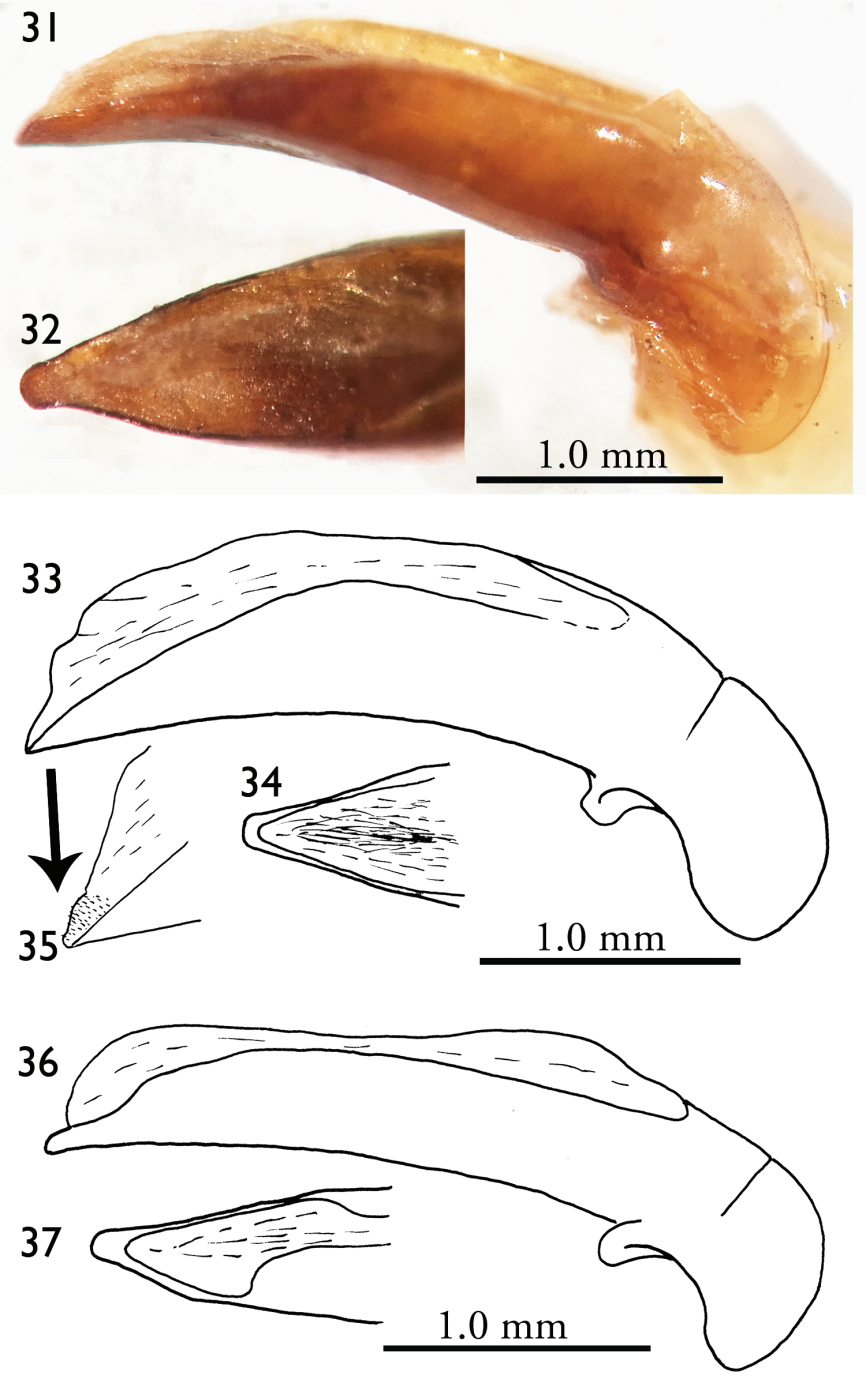
Male genitalia (right lateral view and dorsal view for apical lamella) **31–32** *Orthogonius aberlenci* sp. n. **33–35** *Orthogonius bellus* sp. n. **36–37** *Orthogonius freyi*.

**Figures 38–43.**
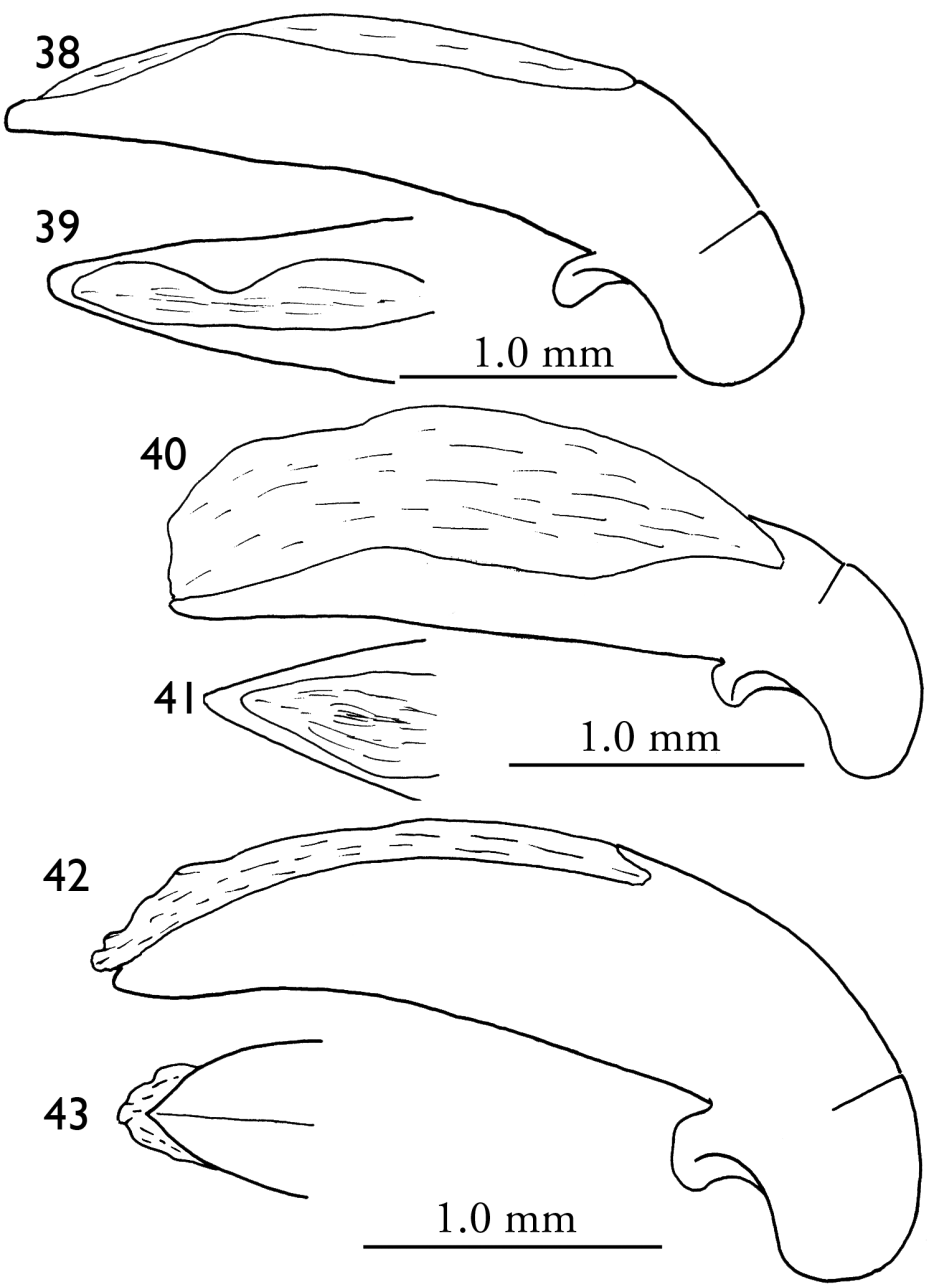
Male genitalia (right lateral view and dorsal view for apical lamella) **38–39** *Orthogonius duboisi* **40–41** *Orthogonius meghalayaensis* sp. n. **42–43** *Orthogonius wrasei* sp. n., right lateral view and ventral view.

####### Remarks.

Separable from other congeners by sex-setose on ventrite VII, deeply emarginate labrum at apical margin and dentate mentum.

####### Etymology.

Referred to the type locality.

####### Distribution.

India.

###### Orthogonius wrasei sp. n.

Taxon classificationAnimaliaColeopteraCarabidae

http://zoobank.org/5A827310-DE68-48D9-B997-B7161BCBA7DC

Figs 3g , 13 , 23 , 42–43

####### Holotype.

Male, “Myanmar (Magway State), nr. Kyeto Pass between Padaukaing-Ngabyin, 850 m, 21.88911 N / 094.41589 E, (secondary forest, lux), 30. VI. 2005, leg. M. Hoffmann & T. Ihle”, “Coll. Wrase BERLIN”, in CDW.

####### Paratypes.

5 females, ibid. in CDW, MNHN and SCAU.

####### Diagnosis.

A black (including lateral expanded pronotal margins) and stout species, having nearly rectangular inner apical angles of elytra, an edentate mentum, a well bordered prosernal process and setose midcoxae.

####### Description.

Length: 18.5–19.0 mm; width: 6.5 mm. Habitus as in Fig. 13.

Body elongate, smooth and shiny. Body black, except palps dark brown. Head and pronotum impunctate, elytral odd intervals with small and sparse punctures; head and pronotum obscurely striate. Microsculptural engraved meshes isodiametric on head and elytra, transverse on pronotum.

Head longer than wide, HL/HW = 1.11–1.13, eyes rather small but prominent, frons and vertex convex, frontal impressions deep, extending beyond level of the fore supraorbital setae; clypeus bisetose, surface almost even; labrum sex-setose, slightly emarginate at apical margin; palps slender, subcylindrical, the 3^rd^ maxillary palpomere as long as the 4^th^ which glabrous, the 3^rd^ with two short setae at apex; the 2^nd^ labial palpomere slightly longer than the 3^rd^, bisetose on inner margin, with several additional setae, the 3^rd^ labial palpomere with a few setae at basal part; ligula small, bisetose at apex; mentum without distinct tooth; each of mentum and submentum bisetose, palpiger asetose. Antennae moderately long, extending over base of elytra; pubescent from basal two fifth of the 4^th^ antennomere, slightly expanded at pubescent portion; the 3^rd^ almost as long as the 4^th^.

Pronotum strongly transverse, PW/PL = 1.76–1.77, disc moderately convex, apical and basal margins well beaded, sides strongly expanded, widest at middle; lateral expanded margins well defined, flat and not reflexed, surface somewhat striate; fore and hind angles broadly rounded; both transversal impressions well marked, basal foveae small and deep.

Elytra elongate ovate, EL/EW = 1.66–1.68, widest at about middle, parallel-sided at middle, basal border complete, apex broadly and obliquely truncated, inner angle nearly rectangular (Fig. 23); striae deep, intervals strongly convex, subequal in width at middle, the 3^rd^ interval with three setiferous pores.

Legs rather slender, fore tibiae slightly expanded at apex, apical margin sinuate, outer angle distinctly protrude, outer margin sub-serrate; middle coxae setose, hind ones smooth and glabrous; middle tibiae quite straight, slightly expanded at apex; hind tibiae slightly expanded at apex, apical spurs long and sharp, the 3^rd^ hind tarsomere longer than the 4^th^ which deeply emarginate at apex (lobes one third as long as the segment); claws strongly pectinate.

Prosternal process well bordered at apex. Ventrite VII quadrisetose in both male and female;

Male genitalia (Figs 42–43): Stout and robust, shallowly sinuate ventrally, dorsal opening very wide, gently and gradually narrowed towards apex, which blunt in lateral view; apical lamella very short, and very sharp in ventral view.

####### Remarks.

Recognized by its black body and nearly rectangular inner apical angles of elytra.

####### Etymology.

This new species is named in honor of David W. Wrase (Berlin).

####### Distribution.

Myanmar.

## Supplementary Material

XML Treatment for Orthogonius jianfengling

XML Treatment for Orthogonius himalayicus

XML Treatment for Orthogonius limbourgi

XML Treatment for Orthogonius politior

XML Treatment for Orthogonius aberlenci

XML Treatment for Orthogonius bellus

XML Treatment for Orthogonius freyi

XML Treatment for Orthogonius duboisi

XML Treatment for Orthogonius meghalayaensis

XML Treatment for Orthogonius wrasei
